# Associations of maternal hemoglobin and its changes with gestational diabetes mellitus: a prospective cohort study

**DOI:** 10.1186/s12937-026-01306-w

**Published:** 2026-03-13

**Authors:** Danmeng Liu, Doudou Zhao, Fuyang Zhao, Li Shan, Xinru Gao, Yang Mi, Pengfei Qu

**Affiliations:** 1 Translational Medicine Center, Northwest Women’s and Children’s Hospital, Xi’an, 710061 Shaanxi China; 2 School of Nursing, Shaanxi University of Chinese Medicine, Xianyang, 712000 Shaanxi China; 3 Department of Gynecology, Northwest Women’s and Children’s Hospital, Xi’an, 710061 Shaanxi China; 4 Department of Medical Ultrasound Center, Northwest Women’s and Children’s Hospital, Xi’an, 710061 Shaanxi China; 5 Department of Obstetrics, Northwest Women’s and Children’s Hospital, Xi’an, 710061 Shaanxi China

**Keywords:** Pregnancy, Hemoglobin, Hemoglobin changes, Gestational diabetes mellitus

## Abstract

**Background:**

Higher iron status during pregnancy may increase gestational diabetes mellitus (GDM) risk via iron-induced oxidative stress, leading to insulin resistance and impairing pancreatic β-cell function through lipid peroxidation and/or DNA damage. However, associations between maternal iron status and GDM remain controversial. Hemoglobin is a clinically routine iron status indicator measured across pregnancy. This study aims to explore whether hemoglobin levels (and changes) in early and mid-pregnancy are associated with GDM risk.

**Methods:**

This study employed longitudinal data from a prospective cohort study conducted from December 2018 to September 2021. Pregnancy women at early pregnancy were recruited in the birth cohort, and 6420 eligible women were included in the final analysis of the present study. Data collected at early and mid-pregnancy was used. Generalized linear models were applied to evaluated the associations between hemoglobin and GDM.

**Results:**

49.6% (3184 of 6420) and 7.1% (458 of 6420) of women had high hemoglobin in early and mid-pregnancy, respectively. 92.6% (5946 of 6420) of women had decreased hemoglobin concentrations from early to mid-pregnancy. The overall incidence of GDM was 23.1% (1484 of 6420). Higher hemoglobin concentrations (for every 10 g/L increase) were associated with increased risk of GDM (early pregnancy: RR: 1.09, 95%CI: 1.04, 1.14; mid-pregnancy: RR: 1.07, 95%CI: 1.01, 1.13). High hemoglobin in early or/and mid-pregnancy significantly increased GDM risk (early pregnancy: RR: 1.11, 95%CI: 1.01, 1.21; mid-pregnancy: RR: 1.19, 95%CI: 1.03, 1.39; both periods: RR: 1.36, 95%CI: 1.15, 1.61). Significantly reduced risk of GDM was observed with every 10 g/L decrease in hemoglobin from early to mid-pregnancy (RR: 0.87, 95%CI: 0.81, 0.93), especially in women with the High-Decrease change pattern (RR: 0.87, 95%CI: 0.80, 0.95).

**Conclusions:**

Maternal high hemoglobin levels in early and/or mid-pregnancy are associated with increased GDM risk, and a decline in hemoglobin levels from early to mid-pregnancy is associated with reduced GDM risk.

**Clinical trial number:**

Not applicable.

**Supplementary Information:**

The online version contains supplementary material available at 10.1186/s12937-026-01306-w.

## Background

Gestational diabetes mellitus (GDM), defined as glucose intolerance of variable severity with onset or first recognition during pregnancy, is one of the most common gestational complications. The global standardized prevalence of GDM stands at 14.0%, with regional variations ranging from 7.1% to 27.6% [1]. In many countries, its prevalence has increased by over 30% within one or two decades, forming a global epidemic trend [2]. GDM not only act as a risk factor for adverse pregnancy and birth outcomes but also correlates with increased risks of chronic diseases later in life [3, 4].

The International Association of Diabetes in Pregnancy Study Group (IADPSG) recommends diagnosing GDM via a 2-hour, 75-g oral glucose tolerance test (OGTT) administered between 24 and 28 weeks of pregnancy. This diagnostic criterion has been adopted by many countries, including China [5]. However, since maternal adaptive metabolic changes in insulin secretion and insulin sensitivity initiate in early pregnancy, GDM onset may occur significantly earlier than 24 weeks of gestation [6, 7]. Exploring early-pregnancy risk factors for GDM is therefore critical for its prediction and prevention.

Iron is an essential and modifiable nutritional factor for maternal and fetal health. Accumulating evidence indicates that iron possibly plays an important role in the progress of GDM [8]. In particular, higher iron stores, elevated iron status, and excessive iron supplementation during pregnancy are potentially associated with an increased risk of GDM [8–10]. While the precise underlying mechanism remains incompletely elucidated, emerging evidence suggests a plausible pathway: excessive iron accumulation enhances reactive oxygen species (ROS) production, thereby exacerbating oxidative stress [11, 12]. Such iron-induced oxidative stress may act through lipid peroxidation and/or DNA damage to subsequently induce abnormal elevation of insulin resistance and impair pancreatic β-cell function, ultimately leading to persistent hyperglycemia and GDM pathogenesis [11, 13].

Despite these mechanistic insights, the clinical association between iron status and GDM remains inconclusive and warrants further investigation. A primary source of inconsistency in previous findings stems from variations in the timing of iron status assessments. Many cross-sectional or case-control studies examining iron biomarkers in mid-pregnancy fail to eliminate reverse causality, as GDM-related metabolic perturbations could themselves alter iron homeostasis [10]. Although prospective studies focusing on early-pregnancy iron status mitigate this limitation, conflicting findings remain—potentially due to small sample sizes and insufficient control of confounding factors [8]. Additionally, given that GDM pathogenesis is a dynamic process, changes in iron status during pregnancy may influence GDM risk; however, longitudinal investigations exploring this relationship remain scarce [14].

Beyond timing-related inconsistencies, the selection of different iron status indicators also contributed to the controversial results [8]. Serum ferritin (SF), a direct biomarker reflecting body iron stores, has been widely used in many studies to assess the association between iron status and GDM. However, SF levels are also susceptible to the effects of inflammation, which can introduce confounding into the observed association between SF and GDM [15]. Additionally, the optimal SF range for reducing GDM risk remains undefined, primarily due to the lack of unified classification thresholds for SF, which ultimately limits its clinical application [10, 16]. In contrast, hemoglobin concentration, another clinically routine indicator reflecting iron status [17], is regularly measured in different pregnancy periods, with standardized criteria for anemia assessment. These advantages provide a consistent classification threshold for interpreting its associations with GDM and allow for longitudinal tracking of iron-related changes during pregnancy, which directly enhance its clinical applicability and feasibility for broader promotion. However, the association between hemoglobin and GDM has been less investigated, and existing evidence remains conflicting [8, 18]. Thus, clarifying the relationship between hemoglobin status and GDM could help address current evidentiary uncertainties and complement the research gaps.

Based on these research gaps, the present study hypothesizes that: (1) maternal hemoglobin levels in early pregnancy and mid-pregnancy are independently associated with GDM risk, with high levels potentially increasing susceptibility; (2) changes in hemoglobin concentrations between early and mid-pregnancy may modify GDM risk. Using a prospective cohort design, this study aims to quantify these associations to provide targeted evidence for optimizing GDM prevention strategies.

## Methods

### Study design and participants

This study employed longitudinal data from a prospective cohort study conducted in Shaanxi Province of Northwest China, between December 2018 and September 2021—a component of the China Birth Cohort Study (CBCS) [19]. Pregnant women were recruited to the birth cohort at the Department of Obstetrics, Northwest Women’s and Children’s Hospital, and followed up until delivery.

The inclusion criteria of participants were (1) Early pregnancy (6 ~ 13^+6^ weeks of gestational age); (2) Plan to receive pregnancy care and deliver at Northwest Women’s and Children’s Hospital; (3) No notifiable infectious diseases (such as hepatitis B, syphilis, and HIV); (4) Ability to understand the study, voluntary participation in the birth cohort, and provision of signed informed consent. The exclusion criteria were: (1) Aged < 20 years; (2) Inability to answer questions accurately due to psychiatric conditions or other serious illnesses. Termination or withdrawal criteria included: (1) Development of serious diseases or death from uncontrollable factors during the observation period; (2) Request for withdrawal or loss to follow-up.

In line with the present study’s objective—investigating the association between maternal hemoglobin levels in early and mid-pregnancy, hemoglobin changes between these two periods, and GDM—participants who met the following criteria were further excluded: (1) Incomplete responses at enrollment; (2) Withdrawal from the study or loss to follow-up prior to OGTT; (3) Failure to complete OGTT; (4) OGTT completion outside the 24 ~ 28 weeks of gestational age window; (5) Incomplete hemoglobin testing in early pregnancy, mid-pregnancy, or both; (6) Mid-pregnancy hemoglobin testing conducted after OGTT; (7) Pre-existing chronic diseases (e.g., hypertension, diabetes mellitus, hyperthyroidism, heart disease, chronic kidney disease, tuberculosis); (8) Gestational hyperglycemia or GDM diagnosed before 20 weeks of gestation; (9) Missing data at enrollment or during follow-up prior to OGTT.

### Data collection

Data was collected at enrollment and during three follow-up assessments. At enrollment (early pregnancy, 6 ~ 13^+6^ weeks), information on sociodemographic characteristics, health status, reproductive history, lifestyle behaviors, and other health-related factors (pre-pregnancy and early pregnancy) was collected via the Enterprise Data Center (EDC) cloud-based online platform. After enrollment, three follow-up assessments were conducted for participants at mid-pregnancy (20 ~ 23^+6^ weeks), late pregnancy (28 ~ 33^+6^ weeks), and post-delivery (within 1 week after delivery). Data on maternal and fetal health status between consecutive follow-ups were extracted from the hospital’s outpatient electronic medical record system, while pregnancy outcomes were obtained from the hospital’s inpatient electronic medical record system. All data were recorded in the EDC system. Clinical laboratory measurements during pregnancy were also extracted from the hospital’s electronic medical record system. For the present study, only data collected at early and mid-pregnancy were used.

### Sample size

Sample size was calculated based on the primary outcome (GDM). Assuming the incidence of GDM was 18% in the non-high hemoglobin concentration group (< 130 g/L) and 26% in the high hemoglobin group (≥ 130 g/L) [20], with a two-sided significance level of 5% and statistical power of 80%, the minimum estimated sample size was 662 participants (331 per group). Our study included 6420 women in the final analysis, which fully met the required sample size.

### Hemoglobin measurement

Hemoglobin concentrations were recorded based on test results extracted from the hospital’s electronic medical record system at enrollment and during follow-up assessments. Gestational age at enrollment and antenatal visits was calculated using the date of the last menstrual period (LMP). For the present study, only women with hemoglobin records in both early pregnancy (10 ~ 13^+6^ weeks; extended to 14^+ 6^ weeks) and mid-pregnancy (20 ~ 27^+6^ weeks; extended to 28^+ 6^ weeks) were included. For women with multiple hemoglobin records within a single pregnancy period, the lowest value was used for analysis [21–23].

Hemoglobin concentration was classified into three groups: anemia (< 110 g/L), normal (110 ~ 129 g/L), and high (≥ 130 g/L). This classification is based on pregnancy-specific physiological changes and evidence-based standards: Normal pregnancy involves plasma volume expansion (peaking at 34 weeks, + 45 ~ 50%) that outpaces red blood cell (RBC) mass increase (+ 20 ~ 30%), leading to adaptive hemodilution [24]. Hb < 110 g/L indicates insufficient RBC mass to compensate for this expansion and can be diagnosed as gestational anemia in accordance with the WHO standard (severity stratified as mild: 100 ~ 109 g/L, moderate: 70 ~ 99 g/L, severe: <70 g/L) [25]. Maternal hemoglobin ≥ 130 g/L is defined as a relatively high concentration during pregnancy according to a meta-analysis [26], which may reflect iron overload or inadequate plasma volume expansion [15, 26, 27].

Absolute changes in hemoglobin were calculated by subtracting early-pregnancy hemoglobin values from mid-pregnancy values. Relative changes in hemoglobin were calculated by dividing the absolute changes by early-pregnancy hemoglobin values and then multiplying by 100%. Hemoglobin changes were classified based on their direction from early to mid-pregnancy (< 0/≥0 g/L). Hemoglobin change patterns were summarized by combing to early-pregnancy hemoglobin status and the direction of hemoglobin change.

### Outcome assessment

The primary outcome of this study was the incidence of GDM. GDM diagnosis was based on the results of a 75 g 2-h OGTT conducted between 24 and 28 weeks of gestation, in accordance with the IADPSG criteria. The diagnostic thresholds for GDM (plasma glucose, mmol/L) are as follows: fasting ≥ 5.1, 1-h ≥ 10.0, and 2-h ≥ 8.5. A diagnosis of GDM was made if any one of these thresholds was exceeded [5].

### Covariate assessment

Based on the existing literature [20, 28–31], covariates considered in the study mainly included two parts: (1) socio-demographic characteristics, including maternal age at registration, education level, occupation, and annual household income. These factors determine the baseline health conditions (e.g., age-related metabolic status) and nutritional intake during pregnancy [32], which were related to hemoglobin levels or GDM risk; (2) health-related characteristics, including parity, pre-pregnancy body mass index (BMI) [33], mode of conception, number of fetuses, menstrual cycle regularity, GDM history, passive smoking, alcohol consumption, folic acid (FA) supplementation, multi-micronutrient (MMN) supplementation, medication consumption, and pregnancy complications. Additionally, Plasma volume expansion during pregnancy influences hemoglobin levels in a predictable pattern across gestational age: it begins to increase early at 6th week, continues to rise by 45–50% till 34th weeks of gestation, and returns to normal within 10–14 days postpartum [24]. Given the unavailability of data on plasma volume expansion in our study, to control for its interference on hemoglobin levels and thereby approximate the actual iron status, we instead adjusted for gestational age at the time of hemoglobin assessment.​ Detailed information of covariate classifications (Table S1) and assessment were described in Supplementary Methods.

### Statistical analysis

Continuous variables were expressed as mean ± SDs, and categorical variables were described as counts (proportions). Comparisons Between-group comparisons were accomplished using t-tests for continuous variables and χ^2^ tests for categorical variables. Hemoglobin change patterns were displayed using a time series plot. The linear associations between hemoglobin status/ changes and GDM were evaluated using generalized linear models (GLMs). Specifically, a binomial distribution with a log link function was used to estimate RRs and their corresponding 95% CIs. Associations between hemoglobin changes and GDM were also estimated stratified by the direction of hemoglobin change or hemoglobin change patterns. Sensitivity analyses were conducted using blood glucose from the OGTT as the outcomes, and also by excluding women with hemoglobin < 100 g/L. Subgroup analyses were applied to assess the association between hemoglobin status/changes according to maternal pre-pregnancy BMI. Interaction analyses were further performed by including an interaction term (hemoglobin status/changes $\times$ pre-pregnancy BMI) in the GLMs to explore potential effect modification.

Additionally, dose-response relationships between hemoglobin concentrations (at the two time points) and GDM were estimated using a restricted cubic spline (RCS) function [34]. According to established cut-offs for anemia [25] and high maternal hemoglobin [26], hemoglobin concentrations of 100, 110, 130 g/L were selected as the three knots in the RCS models. Maternal hemoglobin levels in early or mid-pregnancy were set as the independent variables (x-axis), and GDM was set as the dependent variables (y-axis). The P-value for nonlinear association was used to assess whether a nonlinear association existed between maternal hemoglobin and GDM.

Both unadjusted and adjusted models were constructed for each analysis. The adjusted models were adjusted for propensity scores—estimated using logistic regression (for binary exposures), multiple logistic regression (for multinomial exposures), or GLMs (for continuous exposures) [35]—based on the following covariates: maternal age, education level, occupation, annual household income, parity, pre-pregnancy BMI, mode of conception, number of fetuses, menstrual cycle regularity, GDM history, passive smoking, alcohol consumption, FA supplementation, MMN supplementation, medication consumption, pregnancy complications, and gestational age at the time of hemoglobin assessment. When estimating the association between mid-pregnancy hemoglobin concentration and GDM, an additional adjustment for early-pregnancy hemoglobin concentration was included in the models.

All analyses were performed using SAS version 9.4 (SAS Institute, Cary NC). All statistical tests were two-tailed, and statistical significance was set at *P* < 0.05. GLMs were implemented using the “proc genmod” procedure in SAS; RCS curve fitting was conducted using a SAS macro program developed by Desquilbet [34].

## Results

### Participants for final analyses

**Fig. 1:**
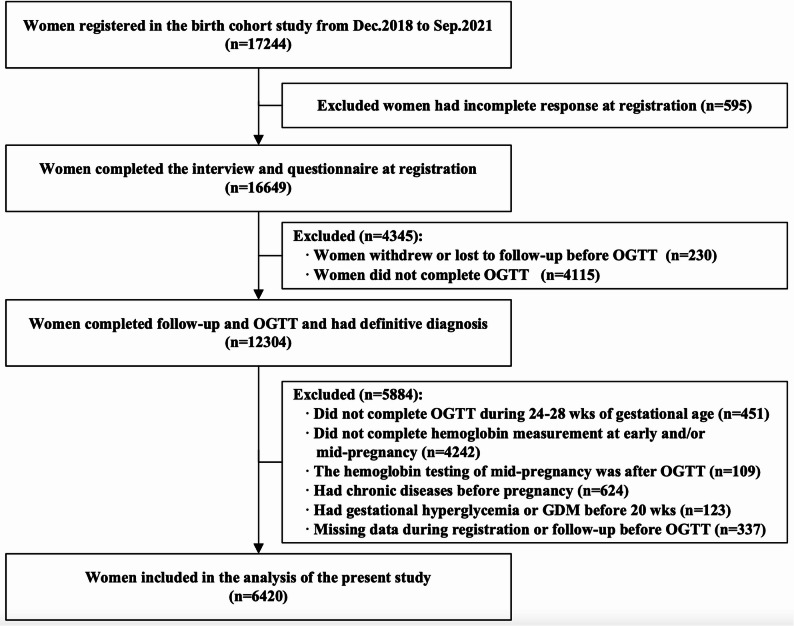
Study flowchart

A total of 17,244 pregnant women enrolled in the birth cohort study between Dec.2018 and Sep.2021. Among these participants, 12,304 were successfully followed up, completed OGTT, and underwent GDM diagnosis. According to the inclusion and exclusion criteria of the present study, 6420 eligible women were included in the final analysis (Fig. 1). Compared with women excluded from the study, included women were younger and had a higher educational level, a greater proportion of employment, and higher household income. They also had a higher proportion of primiparity and natural conception, as well as a lower proportion of pre-pregnancy overweight or obesity and a history of GDM (Table S2).

### Maternal socio-demographic and health-related characteristics

Among the women included in the study, the mean maternal age at enrollment was 29.9 ± 3.4 years. Most women were aged 25–34 years, had a college or university educational level, were employed, were primiparous, had a normal pre-pregnancy weight, and had a regular menstrual cycle (Table 1).

**Table 1:** Description of maternal socio-demographic and health-related characteristics

Characteristics	Mean ± SD or *n* (%)
N	6420
Socio-demographic characteristics	
Age (years)	29.9 ± 3.4
< 25	367 (5.7)
25–34	5555 (86.5)
≥ 35	498 (7.8)
Education	
Senior high school or below	749 (11.7)
College or University	4728 (73.6)
Graduate or above	943 (14.7)
Unemployed or Farmers	1362 (21.2)
Annual household income (RMB, yuan)	
< 100,000	2524 (39.3)
100,000–199,999	2501 (39.0)
≥ 200,000	1395 (21.7)
Health-related characteristics	
Primipara	4642 (72.3)
Pre-pregnancy BMI (kg/m^2^)	
Underweight (< 18.5)	972 (15.1)
Normal weight (18.5–23.9)	4466 (69.6)
Overweight (24.0–27.9)	874 (13.6)
Obesity (≥ 28.0)	108 (1.7)
Nature conceived	6074 (94.6)
Singleton pregnancy	6355 (99.0)
Regular menstrual cycle	5393 (84.0)
Previous GDM	78 (1.2)
Passive smoking ^a^	774 (12.1)
Alcohol consumption ^a^	235 (3.7)
FA supplementation ^a^	6259 (97.5)
MMN supplementation ^a^	3307 (51.5)
Medication consumption ^a^	2412 (37.6)
Pregnancy complications ^b^	562 (8.8)

*BMI* Body mass index, *GDM* Gestational diabetes mellitus, *FA* Folic acid, *MMN* Multiple-micronutrient

^a^Information was collected for the situation during the first trimester

^b^Information was collected for the situation during the first and second trimesters

### Maternal hemoglobin status in different periods and hemoglobin changes between periods

Maternal hemoglobin concentrations were measured at a mean gestational age of 12.8 (SD: 0.7) weeks (early pregnancy) and 26.2 (SD: 0.7) weeks (mid-pregnancy). As displayed in Table 2, the prevalence of high hemoglobin was 49.6% in early pregnancy and 7.1% in mid-pregnancy, respectively. Additionally, 92.6% of women experienced a decrease in hemoglobin levels from early to mid-pregnancy.

**Table 2:** Maternal hemoglobin status in different pregnancy periods and hemoglobin changes between periods

Hemoglobin	Mean ± SD or *n* (%)
At early pregnancy	
Average gestational age at testing (wks)	12.8 ± 0.7
Hemoglobin concentration (g/L)	129.0 ± 9.7
Anemia (< 110)	182 (2.8)
Normal (110–129)	3054 (47.6)
High (≥ 130)	3184 (49.6)
At mid-pregnancy	
Average gestational age at testing (wks)	26.2 ± 1.2
Hemoglobin concentration (g/L)	116.2 ± 9.5
Anemia (< 110)	1431 (22.3)
Normal (110–129)	4531 (70.6)
High (≥ 130)	458 (7.1)
Changes from early to mid-pregnancy	
Absolute changes (g/L)	-12.8 ± 9.1
Relative changes (%)	-9.7 ± 7.2
By change direction	
< 0	5946 (92.6)
Absolute changes (g/L)	-14.3 ± 7.4
Relative changes (%)	-10.9 ± 5.5
≥ 0	474 (7.4)
Absolute changes (g/L)	5.7 ± 7.9
Relative changes (%)	5.4 ± 8.8

### Associations between maternal hemoglobin status in different periods and GDM

The overall incidence of GDM was 23.1% (1484 of 6420 women). A significant increase in GDM risk was observed for every 10 g/L increase in hemoglobin concentration, both in early pregnancy (RR: 1.09, 95%CI: 1.04, 1.14) and mid-pregnancy (RR: 1.07, 95%CI: 1.01, 1.13). Compared with women with normal hemoglobin concentration, those with high hemoglobin concentration in early or mid-pregnancy had a significantly higher risk of GDM (early pregnancy: RR: 1.11, 95%CI: 1.01, 1.21; mid-pregnancy: RR: 1.19, 95%CI: 1.03, 1.39) (Table 3). No non-linear association was observed between maternal hemoglobin concentration in early or mid-pregnancy and GDM (Figure **S1**). When maternal hemoglobin status in both trimesters was considered comprehensively, women with high hemoglobin in both early and mid-pregnancy had a significantly higher GDM risk compared with those with normal hemoglobin in both periods (RR: 1.36, 95%CI: 1.15, 1.61) (Table S3).

Consistent results were observed in the sensitivity analysis examining the relationship between hemoglobin concentration and OGTT-derived plasma glucose levels. For every 10 g/L increase in hemoglobin concentration either in early or mid-pregnancy, significant increases were observed in fasting, 1-h, and 2-h plasma glucose levels. Specifically, compared with women with normal hemoglobin concentrations, those with high hemoglobin concentrations (in early pregnancy, mid-pregnancy, or both) had significantly higher fasting, 1-h, and 2-h plasma glucose levels (**T**ables S4 and S5). Similar results were also observed in the sensitivity analysis that excluded women with hemoglobin concentration < 100 g/L (Tables S6 and S7).

**Table 3:** Associations between maternal hemoglobin status in different pregnancy periods and GDM

Periods	Hemoglobin concentration (g/L)	GDM		
		*n* (%)	Unadjusted model RR (95% CI)	Adjusted model RR (95% CI)
Early pregnancy	Continuous ^a^	1484 (23.1)	1.15 (1.09, 1.20) ^*^	1.09 (1.04, 1.14) ^*^
	Anemia (< 110)	30 (16.5)	0.78 (0.56, 1.09)	0.81 (0.58, 1.13)
	Normal (110–129)	648 (21.2)	Ref.	Ref.
	High (≥ 130)	806 (25.3)	1.19 (1.09, 1.31) ^*^	1.11 (1.01, 1.21) ^*^
Mid-pregnancy	Continuous ^a^	1484 (23.1)	1.13 (1.08, 1.19) ^*^	1.07 (1.01, 1.13) ^*^
	Anemia (< 110)	287 (20.1)	0.86 (0.76, 0.96) ^*^	0.95 (0.84, 1.08)
	Normal (110–129)	1058 (23.4)	Ref.	Ref.
	High (≥ 130)	139 (30.4)	1.30 (1.12, 1.51) ^*^	1.19 (1.03, 1.39) ^*^

GDM Gestational diabetes mellitus; Ref., reference

^a^Estimated RR (95% CI) for GDM with every 10 g/L increase in hemoglobin concentration

### Associations between hemoglobin changes from early to mid-pregnancy and GDM

No association was observed between overall hemoglobin changes and GDM. When the direction of hemoglobin change was considered, among women with a decrease in hemoglobin concentration from early to mid-pregnancy, a significant reduction in GDM risk was observed for every 10 g/L decrease in the absolute change in hemoglobin (RR: 0.87, 95%CI: 0.81, 0.93) and for every 10% decrease in the relative change in hemoglobin (RR: 0.84, 95%CI: 0.77, 0.91) (Table 4).

Six hemoglobin change patterns from early to mid-pregnancy were summarized based on maternal hemoglobin status in early pregnancy and the direction of hemoglobin change by mid-pregnancy. These patterns included: Anemia-Decrease (1.3%), Anemia-Unchanged/Increase (1.5%), Normal-Decrease (42.8%), Normal-Unchanged/Increase (4.8%), High-Decrease (48.5%), and High-Unchanged/Increase (1.1%) (Figure S2). Among women with High-Decrease change pattern, a significant reduction in GDM risk was observed for every 10 g/L decrease in the absolute changes in hemoglobin (RR: 0.87, 95%CI: 0.80, 0.95) or every 10% decrease in the relative changes in hemoglobin (RR: 0.82, 95%CI: 0.73, 0.92) (Table 4).

**Table 4:** Associations between maternal hemoglobin changes from early to mid-pregnancy and GDM

Hemoglobin changes	GDM		
	*n* (%)	Unadjusted RR (95% CI)	Adjusted RR (95% CI)
Overall ^a^	1484 (23.1)		
Absolute changes (g/L)		0.98 (0.94, 1.03)	1.05 (0.99, 1.11)
Relative changes (%)		0.98 (0.92, 1.04)	1.06 (0.99, 1.13)
By change direction			
< 0 ^b^	1399 (23.5)		
Absolute changes (g/L)		0.96 (0.90, 1.02)	0.87 (0.81, 0.93) ^*^
Relative changes (%)		0.92 (0.84, 1.00) ^*^	0.84 (0.77, 0.91) ^*^
≥ 0 ^a^	85 (17.9)		
Absolute changes (g/L)		0.85 (0.63, 1.14)	1.02 (0.71, 1.45)
Relative changes (%)		0.81 (0.60, 1.10)	0.96 (0.67, 1.37)
By hemoglobin change patterns			
Anemia-Decrease ^b^	14 (16.7)		
Absolute changes (g/L)		1.62 (0.90, 2.92)	1.33 (0.68, 2.62)
Relative changes (%)		1.59 (0.88, 2.86)	1.30 (0.67, 2.52)
Anemia-Unchanged/Increase ^a^	16 (16.3)		
Absolute changes (g/L)		0.67 (0.40, 1.13)	0.67 (0.40, 1.12)
Relative changes (%)		0.66 (0.40, 1.10)	0.67 (0.40, 1.10)
Normal-Decrease ^b^	594 (21.6)		
Absolute changes (g/L)		0.94 (0.84, 1.04)	0.91 (0.82, 1.02)
Relative changes (%)		0.91 (0.80, 1.04)	0.89 (0.78, 1.01)
Normal-Unchanged/Increase ^a^	54 (17.6)		
Absolute changes (g/L)		1.09 (0.65, 1.80)	1.12 (0.67, 1.88)
Relative changes (%)		1.06 (0.59, 1.91)	1.10 (0.61, 1.99)
High-Decrease ^b^	791 (25.4)		
Absolute changes (g/L)		0.89 (0.82, 0.97) ^*^	0.87 (0.80, 0.95) ^*^
Relative changes (%)		0.84 (0.75, 0.95) ^*^	0.82 (0.73, 0.92) ^*^
High-Unchanged/Increase ^a^	15 (21.7)		
Absolute changes (g/L)		1.73 (0.37, 8.13)	1.76 (0.37, 8.36)
Relative changes (%)		2.17 (0.28, 17.03)	2.18 (0.28, 17.20)

*GDM* Gestational diabetes mellitus

^a^Estimated RR (95% CI) with every 10 g/L increase in hemoglobin absolute changes or every 10% increase in hemoglobin relative changes

^b^Estimated RR (95% CI) with every 10 g/L decrease in hemoglobin absolute changes or every 10% decrease in hemoglobin relative changes

### Associations between maternal hemoglobin status/changes and GDM according to pre-pregnancy BMI

A higher pre-pregnancy BMI was associated with an increased risk of GDM (RR: 1.10, 95%CI: 1.09, 1.12). As shown in Table 5, significant associations between hemoglobin concentrations (at the two time points) or hemoglobin changes and GDM were primarily observed in women with a normal pre-pregnancy weight. An interaction was observed between pre-pregnancy BMI and early-pregnancy hemoglobin concentration in relation to GDM (P-value for interaction = 0.029).

**Table 5:** Associations between hemoglobin status/changes and GDM by maternal pre-pregnancy BMI

Pre-pregnancy BMI (kg/m^2^)	Hemoglobin status / absolute changes	GDM		
		*n* (%)	Adjusted model RR (95% CI)	*P*_for interaction_^c^
Underweight (< 18.5)	At early pregnancy			0.029
	Anemia (< 110)	5 (11.9)	0.81 (0.35, 1.90)	
	Normal (110–129)	80 (14.7)	Ref.	
	High (≥ 130)	47 (12.2)	0.82 (0.59, 1.15)	
	At mid-pregnancy			0.986
	Anemia (< 110)	33 (12.3)	0.91 (0.63, 1.32)	
	Normal (110–129)	91 (13.9)	Ref.	
	High (≥ 130)	8 (16.7)	1.22 (0.63, 2.35)	
	Absolute changes (g/L)			0.177
	< 0 ^a^	117 (13.4)	1.08 (0.85, 1.37)	
	≥ 0 ^b^	15 (15.6)	1.98 (0.75, 5.26)	
Normal weight (18.5–23.9)	At early pregnancy			
	Anemia (< 110)	21 (16.8)	0.84 (0.56, 1.25)	
	Normal (110–129)	426 (19.9)	Ref.	
	High (≥ 130)	520 (23.7)	1.21 (1.08, 1.35) ^*^	
	At mid-pregnancy			
	Anemia (< 110)	196 (19.4)	0.87 (0.75, 1.00)	
	Normal (110–129)	684 (21.7)	Ref.	
	High (≥ 130)	87 (28.2)	1.29 (1.07, 1.56) ^*^	
	Absolute changes (g/L)			
	< 0 ^a^	913 (22.1)	0.88 (0.81, 0.96) ^*^	
	≥ 0 ^b^	54 (16.6)	0.72 (0.45, 1.16)	
Overweight (24.0–27.9) or obesity (≥ 28.0)	At early pregnancy			
	Anemia (< 110)	4 (26.7)	0.69 (0.30, 1.62)	
	Normal (110–129)	142 (38.8)	Ref.	
	High (≥ 130)	239 (39.8)	1.04 (0.88, 1.22)	
	At mid-pregnancy			
	Anemia (< 110)	58 (38.2)	0.99 (0.79, 1.23)	
	Normal (110–129)	283 (38.8)	Ref.	
	High (≥ 130)	44 (43.6)	1.13 (0.88, 1.43)	
	Absolute changes (g/L)			
	< 0 ^a^	369 (39.7)	0.88 (0.78, 1.00)	
	≥ 0 ^b^	16 (30.2)	1.38 (0.83, 2.30)	

BMI Body mass index; GDM, gestational diabetes mellitus

^a^Estimated RR (95% CI) for GDM with every 10 g/L decrease in hemoglobin absolute changes

^b^Estimated RR (95% CI) for GDM with every 10 g/L increase in hemoglobin absolute changes

^c^P-values for interaction were estimated using the continuous variables of pre-pregnancy BMI and hemoglobin concentrations

## Discussion

In this prospective study, the overall incidence of GDM was 23.1%. The primary findings are as follows: first, GDM risk was associated with higher hemoglobin concentrations in early or mid-pregnancy—rising with increasing hemoglobin levels and being significantly elevated in women with high hemoglobin in either period, with an even higher risk when high levels persisted in both periods. Second, a greater decrease in hemoglobin from early to mid-pregnancy was protective against GDM, and this protective effect was more pronounced in women with the High-Decrease hemoglobin change pattern. Third, the associations between hemoglobin (including both levels in early/mid-pregnancy and dynamic changes across trimesters) and GDM risk were primarily observed in women with a normal pre-pregnancy BMI.

The incidence of high hemoglobin in early pregnancy (49.6%) was higher in our study population than in previously reported cohorts [14, 23]. This discrepancy may be attributed to the more advantaged sociodemographic characteristics of the women included in our study, as higher sociodemographic status is typically associated with better nutritional intake [32], which in turn may elevated the hemoglobin levels. Our findings indicated that high maternal hemoglobin levels in early or mid-pregnancy were associated with an 11% and 19% increased risk of GDM, respectively; notably, the risk was 36% higher when high hemoglobin levels were present in both periods. These results are inconsistent with two previous studies that reported no association between hemoglobin levels (either in mid-pregnancy or both early and mid-pregnancy) and GDM [14, 29]. This inconsistency may be explained by the small sample sizes of those studies, which limited their statistical power to detect significant associations.

In contrast, our findings partially align with a meta-analysis that highlighted the adverse effect of high maternal hemoglobin concentrations before or during the first trimester on GDM risk [26]. However, that meta-analysis did not evaluate the association between hemoglobin levels in the second trimester and GDM, which limits direct comparison with our mid-pregnancy findings. Previous studies have linked high SF levels in the first or second trimester to an elevated risks of GDM [36]. While evidence regarding the association between hemoglobin status and GDM remains limited, our results are consistent with the SF-related findings. This congruence suggests that iron overload may mediate the pathway connecting high hemoglobin levels to an increased risk of GDM.

The possible underlying mechanisms linking iron overload and GDM are as follows. Under normal physiological conditions, iron absorption is tightly regulated by hepcidin—a key hormone controlling iron homeostasis. However, hepcidin expression is partially suppressed during pregnancy [37]. This suppression enhances iron absorption significantly, even in iron-replete women. Excessive iron absorption further elevates SF and hemoglobin levels in these individuals. When iron absorption exceeds the iron-binding capacity of transferrin, circulating non-transferrin-bound iron (NTBI) increases markedly. NTBI is highly reactive and promotes the production of ROS, thereby inducing oxidative stress [11]. This oxidative stress triggers lipid peroxidation and DNA damage in pancreatic β-cells, further hindering insulin synthesis. Concurrently, oxidative stress interferes with glucose metabolism in insulin-sensitive tissues, thereby inducing insulin resistance [15, 27]. Moreover, elevated hemoglobin may increase blood viscosity, while the failure of gestational blood volume expansion to compensate for elevated hemoglobin could further compromise tissue blood flow, reducing glucose uptake in insulin-sensitive tissues and adding another layer of impairment to glucose metabolism [27]. Collectively, these interconnected mechanisms ultimately contribute to the development of GDM.

In our population, 92.6% of women experienced a decrease in hemoglobin levels from early to mid-pregnancy, and most of these women exhibited either the Normal-Decrease or High-Decrease hemoglobin change pattern. We further found that a greater decrease in hemoglobin concentration over this period was associated with a reduced risk of GDM, particularly in women who had high hemoglobin levels in early pregnancy. Our findings provide novel evidence regarding the association between hemoglobin changes and GDM, a relationship that has not been previously reported. This knowledge gap exists because existing studies have not evaluated the association by integrating both early-pregnancy hemoglobin levels and the direction of hemoglobin change [14, 29].

Two potential mechanisms may explain the observed associations between hemoglobin changes and GDM. First, a decrease in hemoglobin concentration may reflect a shift in iron status from high to normal or mild low levels. This shift could mitigate the adverse effects of excessive iron on pancreatic function and glucose metabolism, thereby reducing the risk of developing GDM. Second, maladaptive physiological responses in early pregnancy may result in insufficient plasma volume expansion, placental under-perfusion, or inappropriate fetoplacental signaling, leading to the progression of gestational complications, including GDM [38]. The High-Decrease hemoglobin change pattern observed in our study aligns with the expected physiological adaptations during pregnancy [24]. A greater decrease in hemoglobin from early to mid-pregnancy may indicate adequate plasma volume expansion during this period, which in turn reduces the GDM risk associated with abnormal maternal physiological responses.

Additionally, we observed that women with high hemoglobin levels in early pregnancy, who later had hemoglobin levels within the normal or mildly low range by mid-pregnancy, did not have an increased risk of GDM. Physiologically, pregnancy involves a resetting of the balance between erythrocyte mass and plasma volume. For this reason, mild anemia in the second trimester is generally considered a physiological adaptation rather than a pathological condition, and it does not adversely affect maternal or fetal health [39]. Integrating this physiological background with our study findings, it is reasonable to infer that for women with high hemoglobin status in early pregnancy, lowering hemoglobin concentrations to the normal or mildly low range by mid-pregnancy may be a beneficial adjustment to reduce the GDM risk.

Higher maternal BMI is a well-recognized risk factor for GDM [40]. In our study population, pre-pregnancy BMI modified the association between early-pregnancy hemoglobin concentration and GDM, which was consistent with previous studies [20, 29]. Nevertheless, the association between hemoglobin and GDM was predominantly observed in women with a normal pre-pregnancy BMI. This differs from previous studies, which reported such associations either in women with a pre-pregnancy BMI < 24 kg/m^2^ or in those who were overweight or obese. Obesity contributes critically to GDM development by impairing pancreatic β-cell function, inducing insulin resistance, triggering low-grade chronic inflammation, and disrupting lipid metabolism [28, 40]. Given these potent effects, it is plausible that the influence of pre-pregnancy BMI on GDM risk is more pronounced than that of iron status, and that BMI may act as an effect modifier of iron-related impacts on GDM. However, the underlying mechanisms driving this interaction are complex and require further investigation.

The present study underscores the adverse association between high hemoglobin levels in early and mid-pregnancy and an increased risk of GDM, thereby implying a potential role of excessive iron status in the development of GDM. It further offers valuable insights for clinical practice related to GDM risk management, as hemoglobin—an already routine prenatal test parameter—could provide additional contextual information for assessing GDM risk in pregnant women. A major strength of this study is that it is the first prospective cohort study specifically focusing on the relationship between hemoglobin and GDM, and it also features a relatively large sample size. These characteristics effectively improve the reliability of our results and the validity of conclusions drawn about the association. Additionally, the GDM diagnostic criteria we adopted strictly align with the internationally recognized IADPSG standards [5], and the hemoglobin testing methods utilized are consistent with routine clinical diagnostic practices, ensuring the reliability of the data for analyzing the association between hemoglobin and GDM risk. Furthermore, we meticulously estimated the associations while accounting for a broad range of potential confounders, thereby minimizing the influence of confounding effects. We also employed stratified analyses, interaction analyses, and sensitivity analyses to further reinforce the robustness and reliability of our findings.

Several limitations of the study should be addressed. First, the study population had relatively favorable sociodemographic characteristics, which may limit the generalizability of our findings to more diverse pregnant populations. However, this population still has notable research and clinical value: it consists largely of women with adequate nutritional status, who are more likely to experience high hemoglobin levels in pregnancy. Since our research identified a connection between high hemoglobin levels and higher GDM risk, this group warrants enhanced focus in the assessment of GDM risk. Second, given the unavailability of ferritin data, this study relied solely on hemoglobin for analysis. Hemoglobin levels during pregnancy are influenced by plasma volume expansion, which follows a predictable pattern across gestational age. While we adjusted for gestational age at the time of hemoglobin measurement to indirectly control for potential effects of plasma volume, future research should validate these findings using ferritin and other iron metabolism markers. Notably, our study still offers insights into a potential association between iron status and GDM, which carries certain clinical significance.​ Third, despite many potential confounders were adjusted, some unobserved confounders like genetic background, gestational weight gain, and hyperemesis gravidarum were not able to controlled [15, 41]. Future studies should address these limitations to verify the association between hemoglobin and GDM and explore the underlying mechanisms.

## Conclusions

Maternal high hemoglobin status in early or mid-pregnancy is associated with an increased risk of GDM. A decrease in hemoglobin from early to mid-pregnancy is linked to a potential reduction in GDM risk, particularly in women with high hemoglobin levels in early pregnancy. Further research is warranted to explore the underlying mechanisms linking hemoglobin status and GDM.

## Supplementary Information

Supplementary Material 1: Supplementary Methods: Covariate Assessment. Table S1. Covariates included in the present study and their classifications. Table S2. Comparisons of maternal baseline characteristics between women included and excluded. Table S3. Associations between maternal hemoglobin status in early and mid-pregnancy and GDM. Table S4. Associations between maternal hemoglobin status in different pregnancy periods and blood glucose levels of OGTT. Table S5. Associations between maternal hemoglobin status in different pregnancy periods and blood glucose levels of OGTT. Table S6. Sensitivity analysis: associations between maternal hemoglobin status in different periods and GDM. Table S7. Sensitivity analysis: associations between maternal hemoglobin status in early and mid-pregnancy and GDM. Figure S1. Dose-response relationships between hemoglobin concentrations in A) early pregnancy and B) mid-pregnancy and GDM. Figure S2. Hemoglobin change patterns according to early-pregnancy hemoglobin status and hemoglobin change directions from early to mid-pregnancy.

## Data Availability

The datasets used and/or analysed during the current study are available from the corresponding author on reasonable request.

## Abbreviations

GDM: gestational diabetes mellitus
IADPSG: International Association of Diabetes in Pregnancy Study Group
OGTT: oral glucose tolerance test
ROS: reactive oxygen species
SF: serum ferritin
CBCS: China Birth Cohort Study
EDC: Enterprise Data Center
GLM: generalized linear models
RCS: restricted cubic spline
NTBI: non-transferrin-bound iron
