# Supplementary material for: Associations of maternal hemoglobin and its changes with gestational diabetes mellitus: a prospective cohort study
Source: Nutr J. 2026 Mar 13;25:48. doi: 10.1186/s12937-026-01306-w (PMC13097731; doi:10.1186/s12937-026-01306-w)
Supplement: Supplementary file 1 — Supplementary Material 1: Supplementary Methods: Covariate Assessment. Table S1. Covariates included in the present study and their classifications. Table S2. Comparisons of maternal baseline characteristics between women included and excluded. Table S3. Associations between maternal hemoglobin status in early and mid-pregnancy and GDM. Table S4. Associations between maternal hemoglobin status in different pregnancy periods and blood glucose levels of OGTT. Table S5. Associations between maternal hemoglobin status in different pregnancy periods and blood glucose levels of OGTT. Table S6. Sensitivity analysis: associations between maternal hemoglobin status in different periods and GDM. Table S7. Sensitivity analysis: associations between maternal hemoglobin status in early and mid-pregnancy and GDM. Figure S1. Dose-response relationships between hemoglobin concentrations in A) early pregnancy and B) mid-pregnancy and GDM. Figure S2. Hemoglobin change patterns according to early-pregnancy hemoglobin status and hemoglobin change directions from early to mid-pregnancy. [file 12937_2026_1306_MOESM1_ESM.docx]

**Supplementary Methods**

**Covariate Assessment**

Covariates included in the present study and their classifications are shown in **Table S1**.

**Table S1** Covariates included in the present study and their classifications

| **Covariates** | **Classifications** |
| --- | --- |
| **Socio-demographic characteristics** |  |
| Maternal age at registration (years) | <25/25–34/≥35 |
| Education level | Senior high school or below/college or university/graduate or above |
| Occupation | Employed/unemployed or farmers |
| Annual household income (yuan) | <100,000/100,000–199,999/≥200,000 |
| **Health-related characteristics** |  |
| Parity | Primipara/multipara |
| Pre-pregnancy BMI (kg/m^2^) | Underweight <18.5/normal weight 18.5–23.9/overweight 24.0–27.9/obesity ≥28.0 |
| Mode of conception | Nature conceived/assisted reproductive technique |
| Number of fetuses | Singleton/multiple pregnancy |
| Menstrual cycle regularity | Regular/irregular |
| GDM history | yes/no |
| Passive smoking | yes/no |
| Alcohol consumption | yes/no |
| Folic acid supplementation | yes/no |
| Multi-micronutrient supplementation | yes/no |
| Medication consumption | yes/no |
| Pregnancy complications | yes/no |
| Gestational age at the time of hemoglobin assessment (weeks) | Continuous |

Maternal age was calculated according to birth date. Height and weight were measured to the nearest 0.1 cm and 0.1 kg, respectively. Pre-pregnancy BMI was calculated as weight before pregnancy in kilograms divided by height in meters squared, and was classified according to the standards recommended in the “Guidelines for prevention and control of overweight and obesity in Chinese adults” [1]. Menstrual cycle regularity was reported by women according to their menstrual cycle and duration. GDM history was referred to the diagnosed GDM in the past pregnancy. Passive smoking was defined as being exposed to other people’s cigarettes for more than 15 min/d. Any alcohol drinking in the first trimester was regarded as alcohol consumption. Women who consumed FA or MMN supplement more than 1 week during the first trimester were considered as users of such supplement. Medication consumption was defined as women took medicine during the first trimester, including progesterone, antibiotic, and others. Pregnancy complications referred to diseases occurred during pregnancy before OGTT, including gestational hypertensive disorders and thyroid disorders.

**References**

[1] Wang, Y.; Sun, M.; Xue, H.; Zhao, W.; Yang, X.; Zhu, X.; Zhao, L.; Yang, Y. Understanding the China Blue Paper on Obesity Prevention and Control and policy implications and recommendations for obesity prevention and control in China. Zhonghua Yu Fang Yi Xue Za Zhi. 2019, 53, 875–884.

**Supplementary tables**

**Table S2** Comparisons of maternal baseline characteristics between women included and excluded

| **Characteristics** | **Women included**  **Mean ± SD or n (%)** | **Women excluded ^a^**  **Mean ± SD or n (%)** | **P** |
| --- | --- | --- | --- |
| N | 6420 | 10229 |  |
| Socio-demographic characteristics |  |  |  |
| Age (years) | 29.9 ± 3.4 | 30.1 ± 3.6 | < 0.001 |
| < 25 | 367 (5.7) | 596 (5.8) | < 0.001 |
| 25 - 34 | 5555 (86.5) | 8575 (83.8) |  |
| ≥ 35 | 498 (7.8) | 1058 (10.3) |  |
| Education |  |  |  |
| Senior high school or below | 749 (11.7) | 1468 (14.4) | < 0.001 |
| College or University | 4728 (73.6) | 7269 (71.1) |  |
| Graduate or above | 943 (14.7) | 1492 (14.6) |  |
| Unemployed or Farmers | 1362 (21.2) | 2458 (24.0) | < 0.001 |
| Annual household income (RMB, yuan) |  |  | 0.009 |
| < 100,000 | 2524 (39.3) | 4102 (40.1) |  |
| 100,000 – 199,999 | 2501 (39.0) | 3757 (36.7) |  |
| ≥ 200,000 | 1395 (21.7) | 2370 (23.2) |  |
| Health-related characteristics |  |  |  |
| Primipara | 4642 (72.3) | 7079 (69.2) | < 0.001 |
| Pre-pregnancy BMI (kg/m^2^) |  |  | < 0.001 |
| Underweight (< 18.5) | 972 (15.1) | 1448 (14.2) |  |
| Normal weight (18.5 - 23.9) | 4466 (69.6) | 6775 (66.2) |  |
| Overweight (24.0 - 27.9) | 874 (13.6) | 1462 (14.3) |  |
| Obesity (≥ 28.0) | 108 (1.7) | 544 (5.3) |  |
| Natural conception | 6074 (94.6) | 9553 (93.4) | 0.002 |
| Singleton pregnancy | 6355 (99.0) | 10060 (98.4) | < 0.001 |
| Regular menstrual cycles | 5393 (84.0) | 8527 (83.4) | 0.276 |
| Previous GDM | 78 (1.2) | 164 (1.6) | 0.042 |
| BMI, body mass index; GDM, gestational diabetes mellitus.  ^a^ 595 women with missing data on the baseline information were excluded from comparison. | | | |

**Table S3** Associations between maternal hemoglobin status in early and mid-pregnancy and GDM

| **Hemoglobin status** | | |  | **GDM** | | |
| --- | --- | --- | --- | --- | --- | --- |
| **At early pregnancy** | **At mid-pregnancy** | **N (%)** |  | **n (%)** | **Unadjusted model**  **RR (95% CI)** | **Adjusted model**  **RR (95% CI)** |
| Anemia (< 110 g/L) | Anemia (< 110) | 117 (1.8) |  | 19 (16.2) | 0.76 (0.50, 1.15) | 0.76 (0.50, 1.15) |
|  | Normal (110 - 129) | 59 (0.9) |  | 11 (18.6) | 0.87 (0.51, 1.49) | 0.84 (0.49, 1.43) |
|  | High (≥ 130) | 6 (0.1) |  | 0 (0.0) | – | – |
| Normal (110 – 129 g/L) | Anemia (< 110) | 1043 (16.3) |  | 214 (20.5) | 0.96 (0.83, 1.11) | 0.93 (0.81, 1.08) |
|  | Normal (110 - 129) | 1968 (30.7) |  | 422 (21.4) | Ref. | Ref. |
|  | High (≥ 130) | 43 (0.7) |  | 12 (27.9) | 1.30 (0.80, 2.12) | 1.20 (0.74, 1.95) |
| High (≥ 130 g/L) | Anemia (< 110) | 271 (4.2) |  | 54 (19.9) | 0.93 (0.72, 1.20) | 0.89 (0.67, 1.11) |
|  | Normal (110 - 129) | 2504 (39.0) |  | 625 (25.0) | 1.16 (1.04, 1.30) ^*^ | 1.11 (0.99, 1.23) |
|  | High (≥ 130) | 409 (6.4) |  | 127 (31.1) | 1.45 (1.22, 1.71) ^*^ | 1.36 (1.15, 1.61) ^*^ |

**Table S4** Associations between maternal hemoglobin status in different pregnancy periods and blood glucose levels of OGTT ^a^

| **Hemoglobin concentration (g/L)** | **Fasting PG (mmol/L)** | | |  | **1-h PG (mmol/L)** | | |  | **2-h PG (mmol/L)** | | |
| --- | --- | --- | --- | --- | --- | --- | --- | --- | --- | --- | --- |
|  | **mean (SD)** | **Unadjusted model**  **MD (95% CI)** | **Adjusted model**  **MD (95% CI)** |  | **mean (SD)** | **Unadjusted model**  **MD (95% CI)** | **Adjusted model**  **MD (95% CI)** |  | **mean (SD)** | **Unadjusted model**  **MD (95% CI)** | **Adjusted model**  **MD (95% CI)** |
| At early pregnancy |  |  |  |  |  |  |  |  |  |  |  |
| Continuous ^a^ | 4.75 (0.44) | 0.03 (0.02, 0.04) ^*^ | 0.02 (0.00, 0.03) ^*^ |  | 7.53 (1.69) | 0.14 (0.10, 0.19) ^*^ | 0.08 (0.04, 0.13) ^*^ |  | 6.64 (1.29) | 0.13 (0.10, 0.16) ^*^ | 0.09 (0.06, 0.13) ^*^ |
| Anemia (< 110) | 4.70 (0.37) | -0.03 (-0.10, 0.03) | -0.02 (-0.09, 0.04) |  | 7.31 (1.37) | -0.10 (-0.35, 0.15) | -0.07 (-0.32, 0.18) |  | 6.37 (1.07) | -0.18 (-0.37, 0.01) | -0.16 (-0.35, 0.03) |
| Normal (110 - 129) | 4.73 (0.43) | Ref. | Ref. |  | 7.41 (1.67) | Ref. | Ref. |  | 6.55 (1.24) | Ref. | Ref. |
| High (≥ 130) | 4.78 (0.44) | 0.05 (0.03, 0.08) ^*^ | 0.03 (0.01, 0.05) ^*^ |  | 7.66 (1.71) | 0.25 (0.16, 0.33) ^*^ | 0.16 (0.08, 0.24) ^*^ |  | 6.74 (1.34) | 0.18 (0.12, 0.25) ^*^ | 0.13 (0.06, 0.19) ^*^ |
| At mid-pregnancy |  |  |  |  |  |  |  |  |  |  |  |
| Continuous ^a^ | 4.75 (0.44) | 0.02 (0.01, 0.03) ^*^ | 0.01 (-0.00, 0.02) |  | 7.53 (1.69) | 0.17 (0.13, 0.22) ^*^ | 0.12 (0.08, 0.17) ^*^ |  | 6.64 (1.29) | 0.15 (0.11, 0.18) ^*^ | 0.12 (0.08, 0.15) ^*^ |
| Anemia (< 110) | 4.73 (0.40) | -0.02 (-0.05, 0.01) | -0.01 (-0.04, 0.01) |  | 7.34 (1.58) | -0.21 (-0.31, -0.11) ^*^ | -0.16 (-0.26, -0.06) ^*^ |  | 6.47 (1.18) | -0.19 (-0.27, -0.11) ^*^ | -0.16 (-0.24, -0.08) ^*^ |
| Normal (110 - 129) | 4.75 (0.44) | Ref. | Ref. |  | 7.55 (1.70) | Ref. | Ref. |  | 6.66 (1.31) | Ref. | Ref. |
| High (≥ 130) | 4.83 (0.48) | 0.07 (0.03, 0.12) ^*^ | 0.07 (0.03, 0.12) ^*^ |  | 7.95 (1.81) | 0.40 (0.23, 0.56) ^*^ | 0.40 (0.24, 0.56) ^*^ |  | 6.95 (1.38) | 0.29 (0.16, 0.41) ^*^ | 0.29 (0.17, 0.41) ^*^ |
| PG, plasma glucose; MD, mean difference; Ref., reference.  ^a^ Estimated MD (95% CI) for blood glucose of OGTT with every 10 g/L increase in hemoglobin concentration. | | | | | | | | | | | |

**Table S5** Associations between maternal hemoglobin status in different pregnancy periods and blood glucose levels of OGTT ^a^

| **Hemoglobin status (g/L)** | | **Fasting PG (mmol/L)** | |  | **1-h PG (mmol/L)** | |  | **2-h PG (mmol/L)** | |
| --- | --- | --- | --- | --- | --- | --- | --- | --- | --- |
| **At early pregnancy** | **At mid-pregnancy** | **mean (SD)** | **Adjusted model ^c^**  **MD (95% CI)** |  | **mean (SD)** | **Adjusted model ^c^**  **MD (95% CI)** |  | **mean (SD)** | **Adjusted model ^c^**  **MD (95% CI)** |
| Anemia (< 110 g/L) | Anemia (< 110) | 4.68 (0.37) | -0.04 (-0.12, 0.04) |  | 7.23 (1.38) | -0.23 (-0.54, 0.09) |  | 6.29 (1.06) | -0.30 (-0.53, -0.06) ^*^ |
|  | Normal (110 - 129) | 4.72 (0.36) | -0.01 (-0.13, 0.10) |  | 7.47 (1.41) | -0.01 (-0.44, 0.43) |  | 6.53 (1.12) | -0.07 (-0.40, 0.26) |
|  | High (≥ 130) | 4.63 (0.31) | -0.13 (-0.48, 0.21) |  | 7.26 (0.94) | -0.28 (-1.63, 1.06) |  | 6.41 (0.39) | -0.22 (-1.25, 0.80) |
| Normal (110 – 129 g/L) | Anemia (< 110) | 4.74 (0.41) | 0.01 (-0.02, 0.04) |  | 7.34 (1.60) | -0.11 (-0.24, 0.02) |  | 6.49 (1.19) | -0.09 (-0.19, 0.00) |
|  | Normal (110 - 129) | 4.72 (0.45) | Ref. |  | 7.44 (1.69) | Ref. |  | 6.58 (1.27) | Ref. |
|  | High (≥ 130) | 4.80 (0.41) | 0.07 (-0.06, 0.20) |  | 7.95 (1.93) | 0.48 (-0.03, 0.98) |  | 6.99 (1.11) | 0.39 (0.01, 0.78) ^*^ |
| High (≥ 130 g/L) | Anemia (< 110) | 4.74 (0.39) | 0.02 (-0.04, 0.07) |  | 7.39 (1.57) | -0.11 (-0.32, 0.11) |  | 6.47 (1.17) | -0.14 (-0.30, 0.03) |
|  | Normal (110 - 129) | 4.78 (0.44) | 0.04 (0.02, 0.07) ^*^ |  | 7.64 (1.71) | 0.16 (0.06, 0.26) ^*^ |  | 6.73 (1.34) | 0.13 (0.05, 0.21) ^*^ |
|  | High (≥ 130) | 4.83 (0.49) | 0.09 (0.04, 0.14) ^*^ |  | 7.96 (1.81) | 0.46 (0.28, 0.64) ^*^ |  | 6.95 (1.42) | 0.34 (0.21, 0.48) ^*^ |
| PG, plasma glucose; SD, standard deviation; MD, mean difference; CI, confident interval; Ref., reference. | | | | | | | | | |

**Table S6** Sensitivity analysis: associations between maternal hemoglobin status in different periods and GDM ^a^

| **Hemoglobin concentration (g/L)** | **GDM** | | |
| --- | --- | --- | --- |
|  | **n (%)** | **Unadjusted model**  **RR (95% CI)** | **Adjusted model**  **RR (95% CI)** |
| At early pregnancy |  |  |  |
| Continuous ^b^ | 1431 (23.4) | 1.013 (1.008, 1.018) ^*^ | 1.08 (1.03, 1.14) ^*^ |
| Anemia (< 110) | 16 (17.2) | 0.80 (0.51, 1.26) | 0.82 (0.53, 1.29) |
| Normal (110 - 129) | 611 (21.5) | Ref. | Ref. |
| High (≥ 130) | 804 (25.4) | 1.18 (1.08, 1.30) ^*^ | 1.10 (1.00, 1.20) ^*^ |
| At mid-pregnancy |  |  |  |
| Continuous ^b^ | 1431 (23.4) | 1.013 (1.008, 1.019) ^*^ | 1.10 (1.04, 1.16) ^*^ |
| Anemia (< 110) | 236 (20.6) | 0.88 (0.78, 0.99) ^*^ | 0.95 (0.83, 1.08) |
| Normal (110 - 129) | 1056 (23.4) | Ref. | Ref. |
| High (≥ 130) | 139 (30.7) | 1.31 (1.13, 1.52) ^*^ | 1.20 (1.03, 1.41) ^*^ |
| ^a^ Women with hemoglobin < 100 g/L were excluded. N=6110.  ^b^ Estimated RR (95% CI) for GDM with every 10 g/L increase in hemoglobin concentration. | | | |

**Table S7** Sensitivity analysis: associations between maternal hemoglobin status in early and mid-pregnancy and GDM ^a^

| Hemoglobin status | | |  | GDM | | |
| --- | --- | --- | --- | --- | --- | --- |
| At early pregnancy | At mid-pregnancy | N (%) |  | n (%) | Unadjusted model  RR (95% CI) | Adjusted model  RR (95% CI) |
| Anemia (< 110 g/L) | Anemia (< 110) | 54 (0.9) |  | 7 (13.0) | 0.60 (0.30, 1.21) | 0.61 (0.30, 1.22) |
|  | Normal (110 - 129) | 38 (0.6) |  | 9 (23.7) | 1.10 (0.62, 1.97) | 1.06 (0.59, 1.88) |
|  | High (≥ 130) | 1 (0.0) |  | 0 (0.0) | – | – |
| Normal (110 – 129 g/L) | Anemia (< 110) | 835 (13.7) |  | 177 (21.2) | 0.99 (0.85, 1.16) | 0.97 (0.83, 1.13) |
|  | Normal (110 - 129) | 1968 (32.2) |  | 422 (21.4) | Ref. | Ref. |
|  | High (≥ 130) | 43 (0.7) |  | 12 (27.9) | 1.30 (0.80, 2.12) | 1.19 (0.73, 1.94) |
| High (≥ 130 g/L) | Anemia (< 110) | 258 (4.2) |  | 52 (20.2) | 0.94 (0.73, 1.22) | 0.87 (0.68, 1.13) |
|  | Normal (110 - 129) | 2504 (41.0) |  | 625 (25.0) | 1.16 (1.04, 1.30) ^*^ | 1.10 (0.99, 1.23) |
|  | High (≥ 130) | 409 (6.7) |  | 127 (31.1) | 1.45 (1.22, 1.71) ^*^ | 1.36 (1.15, 1.61) ^*^ |
| ^a^ Women with hemoglobin < 100 g/L were excluded. N=6110. | | | | | | |

**Figure S1** Dose-response relationships between hemoglobin concentrations in A) early pregnancy and B) mid-pregnancy and GDM. Restricted cubic spline functions with three knots (including hemoglobin at 100, 110, 130 g/L) were applied. Models were adjusted for the propensity score that was calculated based on the covariates. The minimum values of hemoglobin at the two periods were set as the reference values. Dashed lines represent the 95% CIs, and knots were displayed by dots. The horizontal dashed green line represents the RR for GDM was 1.00.


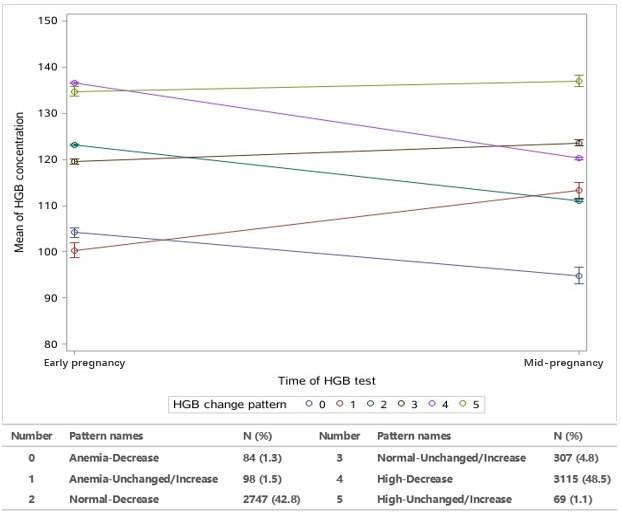


**Figure S2** Hemoglobin change patterns according to early-pregnancy hemoglobin status and hemoglobin change directions from early to mid-pregnancy (< 0 or ≥ 0 g/L). Six patterns were summarized, including Anemia-Decrease (blue line), Anemia-Unchanged/Increase (red line), Normal-Decrease (green line), Normal- Unchanged/Increase (brown line), High-Decrease (purple line), and High- Unchanged/Increase (light green line). The small circles and short lines at both end of each line displayed the mean and 95% CI of hemoglobin concentration in the two periods of each change pattern.
